# Stomata variation in the process of polyploidization in Chinese chive (*Allium tuberosum*)

**DOI:** 10.1186/s12870-023-04615-y

**Published:** 2023-11-28

**Authors:** Peng-Qiang Yao, Jian-Hua Chen, Pei-Fang Ma, Li-Hua Xie, Shi-Ping Cheng

**Affiliations:** 1 Henan Key Laboratory of Germplasm Innovation and Utilization of Eco-Economic Woody Plant, Pingdingshan University, Pingdingshan, 467000 China; 2 Pingdingshan Academy of Agricultural Sciences/Henan Chinese Chive Engineering Technology Research Center, Pingdingshan, 467001 China

**Keywords:** Evolution, Stomatal variation, Polyploidization, Chinese chive

## Abstract

**Background:**

Stomatal variation, including guard cell (GC) density, size and chloroplast number, is often used to differentiate polyploids from diploids. However, few works have focused on stomatal variation with respect to polyploidization, especially for consecutively different ploidy levels within a plant species. For example, *Allium tuberosum*, which is mainly a tetraploid (2n = 4x = 32), is also found at other ploidy levels which have not been widely studied yet.

**Results:**

We recently found cultivars with different ploidy levels, including those that are diploid (2n = 2x = 16), triploid (2n = 3x = 24), pseudopentaploid (2n = 34–42, mostly 40) and pseudohexaploid (2n = 44–50, mostly 48). GCs were evaluated for their density, size (length and width) and chloroplast number. There was no correspondence between ploidy level and stomatal density, in which anisopolyploids (approximately 57 and 53 stomata/mm^2^ in triploid and pseudopentaploid, respectively) had a higher stomatal density than isopolyploids (approximately 36, 43, and 44 stomata/mm^2^ in diploid, tetraploid and pseudohexaploid, respectively). There was a positive relationship between ploidy level and GC chloroplast number (approximately 44, 45, 51, 72 and 90 in diploid to pseudohexaploid, respectively). GC length and width also increased with ploidy level. However, the length increased approximately 1.22 times faster than the width during polyploidization.

**Conclusions:**

This study shows that GC size increased with increasing DNA content, but the rate of increase differed between length and width. In the process of polyploidization, plants evolved longer and narrower stomata with more chloroplasts in the GCs.

**Supplementary Information:**

The online version contains supplementary material available at 10.1186/s12870-023-04615-y.

## Background

Stomata have attracted the attention of scientists for a long time due to their significance in plant productivity. A great deal of knowledge related to the morphology, structure, development, and physiology of stomata has been collected [1–3]. Nevertheless, there are still many unanswered questions regarding the origin and evolution of stomata in plants [4, 5]. To explore the problems of stomatal origin, some scholars have studied the stomata of plant fossil materials [6]. However, the available fossil material is limited and can only explore the divergence of bryophytes and tracheophytes [7]. For the evolution of stomata in plants, some findings have shown that the appearance of stomata is a special structure formed by the pressure of changing environment [8, 9]. Some scholars believe that stomata are ancient structures that had diversified by the Early Devonian [5, 10]. In the process of plant evolution, polyploidization is an important evolutionary feature. Many studies have shown that most green plants, especially flowering plants have one or more events of whole genome duplication in their ancestry [11–13]. However, few works have focused on stomatal morphological variation in the process of polyploidization. We believe that research stomatal variation during plant polyploidization has important reference value for the study of stomatal origin and evolution.

At present, stomatal morphological variation studies between diploids and polyploids mainly include the density per unit area of epidermis, GC size and number of chloroplasts in GCs [14–16]. Polyploidization is considered an important mechanism of plant adaptation to the changing environment by these studies. Because of these morphological variations many physiological functions of plants were affected in polyploids. For example, polyploids use soil water more efficiently, resulting in higher drought resistance [17, 18]. There are differences in CO_2_ use efficiency between diploids and polyploids, which is of great significance for how to select better adapted crops in today’s increasing greenhouse effect [19]. Therefore, understanding stomatal changes in polyploids could have broader implications for plant adaptation and evolution.

In many plant species, stomatal density has often been used as a morphological marker for identifying ploidy level [20, 21]. A negative relation was observed between the stomatal density and ploidy level. However, in some species, such as *Hibiscus syriacus*, stomatal density cannot be used as an indicator to estimate ploidy level [22]. The number of chloroplasts in GCs has also generally been used as a marker for estimating the ploidy level of a given plant. There is a positive relationship between genome size and chloroplast number [23–25]. GC size has also been used as a characteristic to estimate the ploidy level of many plants. GC length has been shown to have a positive correlation with genome size [26, 27]. However, little attention has been given to GC width in plants with different ploidy levels [28].

*Allium tuberosum* is a perennial herbaceous plant species belonging to the Liliaceae family. This species is commonly called Chinese chive and is cultivated on a large scale in China, Japan, Korea, Vietnam and India [29]. It is used as a vegetable as well as a spice. It is also used as a cure for hypertension due to its antimicrobial and carminative properties [30]. Almost all Chinese chive varieties and landraces are autotetraploids with a chromosome number of 2n = 4x = 32 [13] and display a high degree of diplosporous apomixis with pseudogamous seed development [31]. Except in the case of tetraploids, different ploidy levels of Chinese chive, such as diploidy [29] and hexaploidy [32], have been discovered. These findings suggested that the reproduction of Chinese chives may vary, addition to parthenogenesis. At present, few works have focused on exploring the different ploidy levels of Chinese chives, and no further development and utilization of these materials has been conducted.

In this study, 420 cultivars were used as materials to screen individuals with different ploidy levels. Flow cytometry combined with chromosome counting was used to determine the ploidy level. Stomal characteristics, including the density, size (length and width) and chloroplast number in the GCs, were observed and measured. Stomatal variation in the evolutionary process of polyploidization was explored and discussed.

## Results

### Estimation of ploidy levels of each cultivar by flow cytometry

**Fig. 1:**
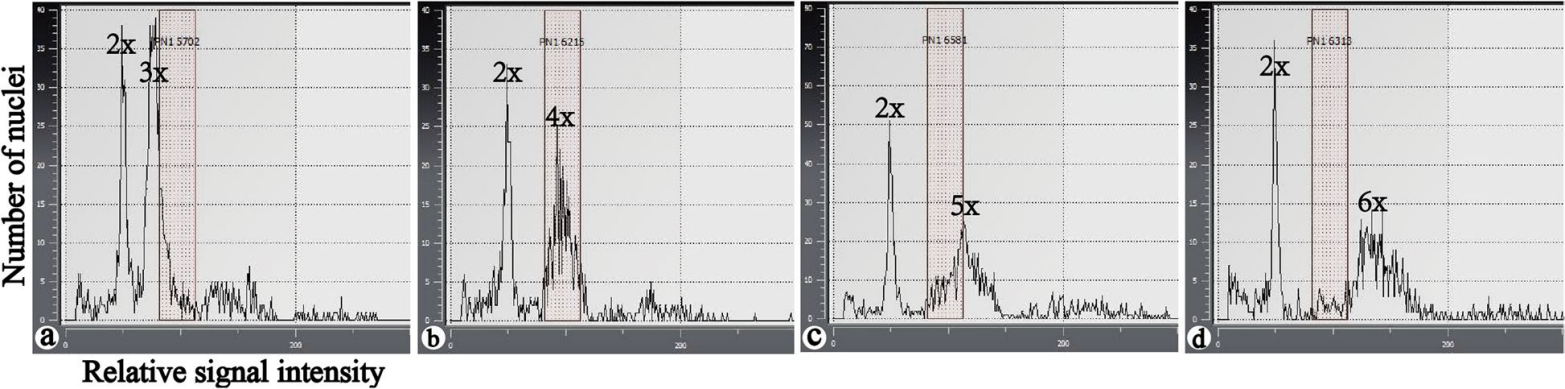
Ploidy level detection of the Chinese chive by flow cytometry (the x- and y-axes represent the relative signal intensity and number of nuclei, respectively). **a** Diploids and triploids. **b** Diploids and tetraploids. **c** Diploids and pentaploids. **d** Diploids and hexaploids

Ploidy levels were inferred by flow cytometry analyses, as shown in Fig. 1. The results showed that 10 individuals of one cultivar that were chosen randomly had the same ploidy level. Among them, one diploid (as the internal reference, Fig. 1a-d), two triploid (Fig. 1a), one pentaploid (Fig. 1c), one hexaploid (Fig. 1d) and 415 tetraploid cultivars were screened (Fig. 1b). Compared with the cultivars with the other ploidy levels, the pentaploid and hexaploid cultivars exhibited a wider and lower peak according to the data.

### Determination of ploidy level by chromosome counting

**Fig. 2:**
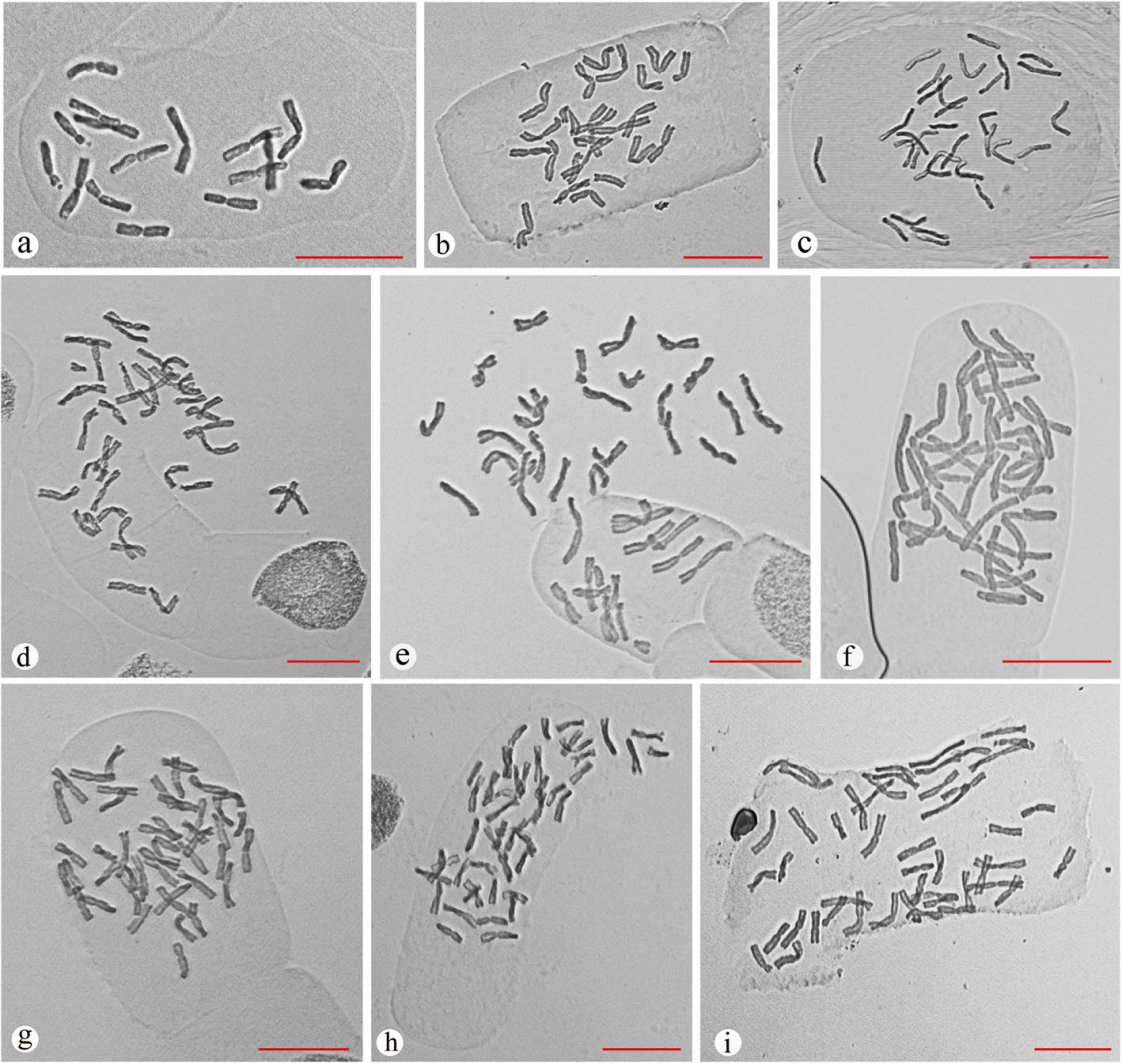
Different ploidy levels and chromosome numbers of Chinese chives (scale bar = 20 μm). **a** Diploid chromosome number (2n = 2x = 16). **b** Triploid chromosome number (2n = 3x = 24). **c** Tetraploid chromosome number (2n = 4x = 32). **d-f** Pentaploid chromosome number (2n = 34, 40, and 42). **g-i** Hexaploid chromosome number (2n = 44, 46, and 48)

Chromosome counts showed that the diploid chromosome number was 2n = 2x = 16 (Fig. 2a), the triploid chromosome number was 2n = 3x = 24 (Fig. 2b), and the tetraploid chromosome number was 2n = 4x = 32 (Fig. 2c). The pentaploids had 34–42 chromosomes, most of which had 40 chromosomes (Fig. 2d-f). The hexaploids had 44–50 chromosomes, and most had 48 chromosomes (Fig. 2g-i). Given these facts, we named the pentaploids and hexaploids pseudopentaploids and pseudohexaploids, respectively. The chromosome counting results corresponded to the flow cytometry detection results.

**Fig. 3:**
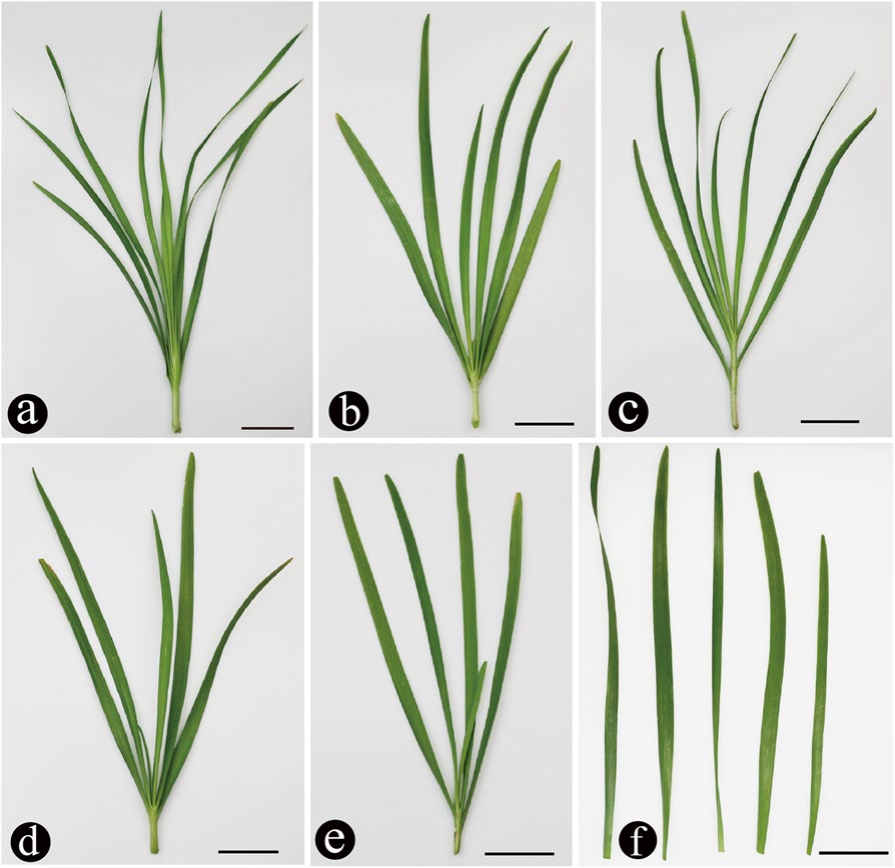
Morphological characters of Chinese chives with different ploidy levels (scale bar = 5 cm). **a** Diploid. **b** Triploid. **c** Tetraploid. **d** Pseudopentaploid. **e** Pseudohexaploid. **f** Comparison of leaves of different ploidy levels. From left to right are diploid, triploid, tetraploid, pseudopentaploid and pseudohexaploid

The Chinese chive diploid is characterized by a harder, erect and twisted leaf blade (Fig. 3a). In our study, two triploids, which had wide and long leaf characteristics, were screened (Fig. 3b). Indeed, tetraploids have abundant external characteristics, but most are similar to those shown in Fig. 3c. In this study, we mainly chose cultivar 791 as the representative tetraploid, which is planted largely in China. Compared with the cultivars with other ploidy levels, the pseudopentaploids and pseudohexaploids seem to have thicker leaves (Fig. 3d and e). For these cultivars, except for the diploids, the cultivars with different ploidy levels cannot be distinguished from each other by morphological characteristics alone (Fig. 3f).

### Comparison of stomatal density among different ploidy levels of Chinese chive

**Fig. 4:**
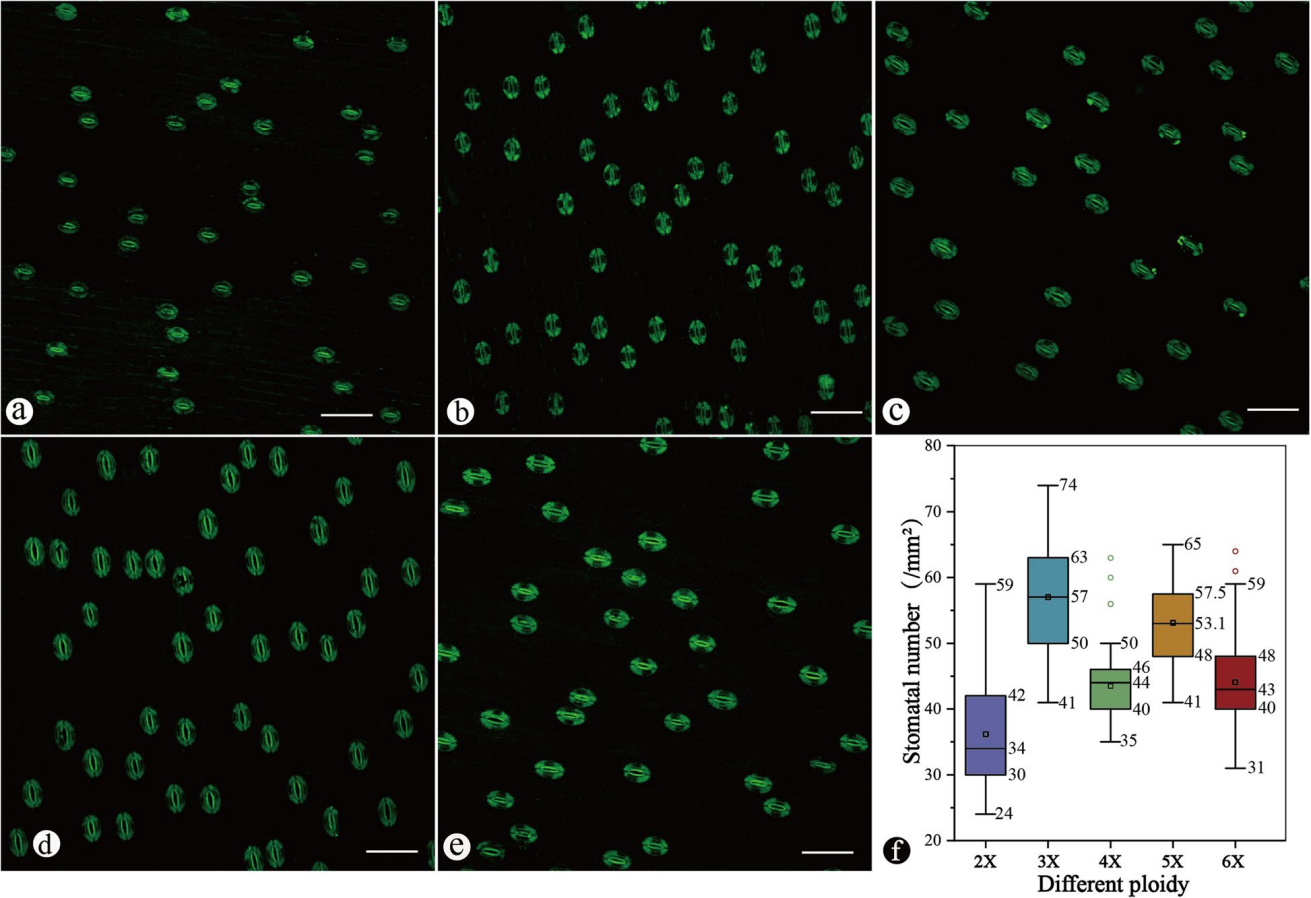
Epidermal layer of Chinese chive showing the stomatal number under fluorescent light (scan area = 850.2 μm × 850.2 μm). **a** Diploid. **b** Triploid. **c** Tetraploid. **d** Pseudopentaploid. **e** Pseudohexaploid. **f** Boxplots of stomatal number to compare different ploidy levels of Chinese chive

**Fig. 5:**
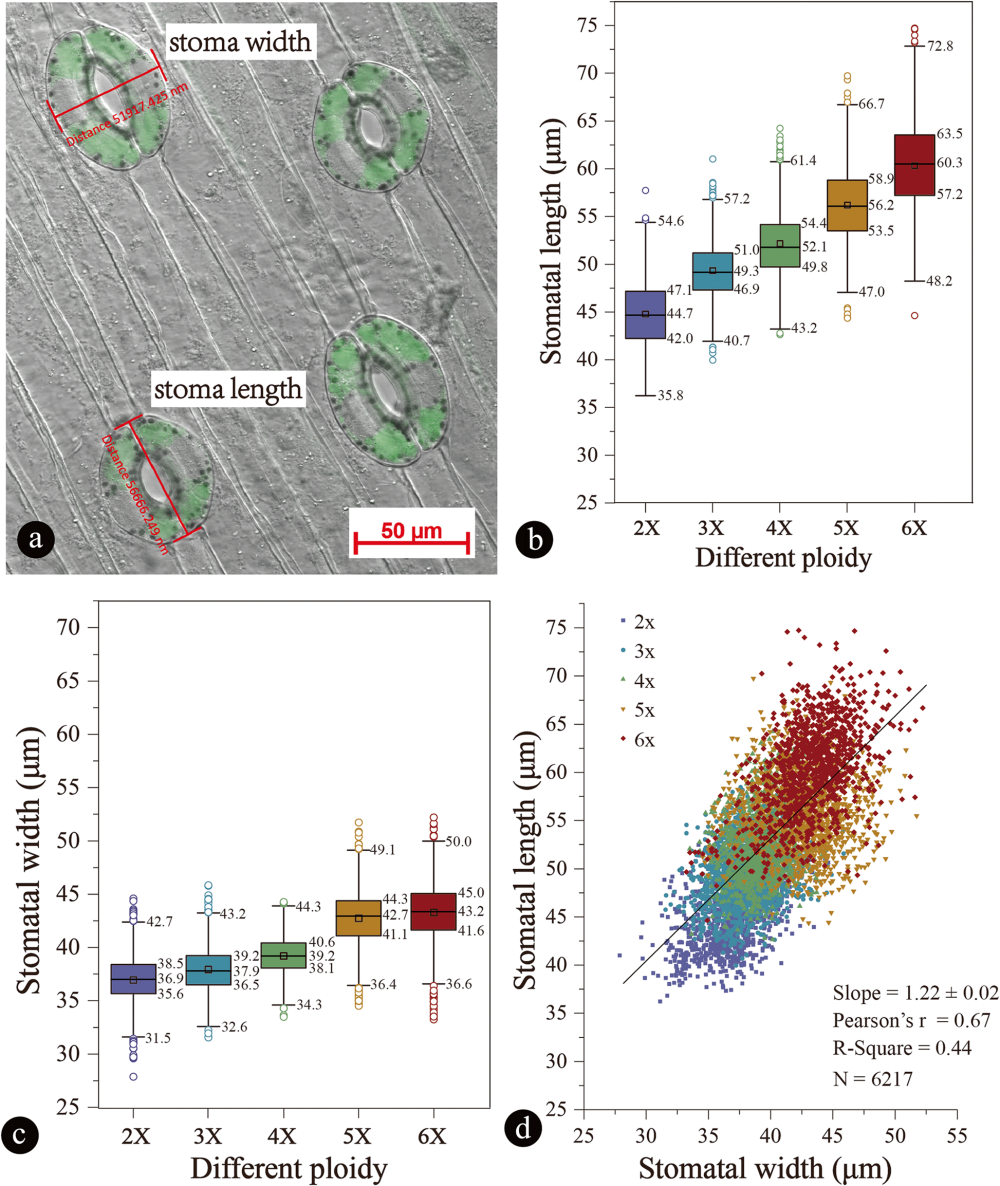
Stomal length and width of Chinese chive cultivars with different ploidy levels. **a** Stomatal length—measured in the longitudinal stoma axis; stomatal width—measured as the largest distance found between outer guard cell walls perpendicular to the longitudinal stomatal axis. **b** Boxplots of stomatal length to compare different ploidy levels of Chinese chive. **c** Boxplots of stomatal width to compare different ploidy levels of Chinese chive. **d** Comparison between length and width of GCs in the process of polyploidization

The stomata of the Chinese chive consisted of two similarly sized cells that were shaped like the kidneys (Fig. 4a-e, Fig. 5a). The average number of stomata per unit area of the diploid (36.16 ± 8.09 stomata/mm^2^) was lower than that of the others (Fig. 4a), while the triploids had the highest density of stomata (57.00 ± 8.11 stomata/mm^2^) (Fig. 4b). The results showed that anisopolyploid plants, such as triploid and pseudopentaploid plants (53.11 ± 5.99 stomata/mm^2^), had a higher density than isopolyploid plants, such as diploid (36.16 ± 8.09 stomata/mm^2^), tetraploid (43.49 ± 5.13 stomata/mm^2^) and pseudohexaploid (44.08 ± 6.83 stomata/mm^2^) plants (Fig. 4f). Comparison analysis suggested that the density of GCs differed markedly among different ploidy levels of Chinese chives (*p* = *0.000*). Multiple comparison analysis suggested that there was no significant difference between tetraploids and pseudohexaploids in stomatal number per area, which differed markedly from those of the other cultivars with different ploidy levels.

### Comparison of length and width of GCs among Chinese chives of different ploidy levels

The stomatal length and width data are shown in Fig. 5a. The results showed that GCs of different sizes were present within a cultivar. In general, the GC length and width of different cultivars showed a positive correlation with ploidy level (Fig. 5b and c), suggesting the possibility of using this method to distinguish ploidy levels in Chinese chives. Stomatal length and width increased as the ploidy level increased, although there were some overlaps of their values among the different ploidy levels. The increasing trend was more obvious for the length than for the width (Fig. 5b and c). The increase in length was approximately 1.22 times faster than that of the width in the process of polyploidization (Fig. 5d).

In these cultivars, the shortest GC length was 36.223 μm in the diploids, and the longest was 74.727 μm in the pseudohexaploids. The average GC lengths from small to large were 44.799 ± 3.293 μm (diploid), 49.319 ± 3.000 μm (triploid), 52.151 ± 3.476 μm (tetraploid), 56.193 ± 3.947 μm (pseudopentaploid) and 60.325 ± 4.706 μm (pseudohexaploid). The results of our comparative analysis suggested that the length of the stomata differed markedly among Chinese chive cultivars with different ploidy levels.

The variation trend of the GC width was the same as that of the length. The shortest occurred for the diploids (36.935 ± 2.369 μm), and the longest occurred for the pseudohexaploids (43.281 ± 2.756 μm), followed by the triploids (37.929 ± 2.090 μm), tetraploids (39.213 ± 1.813 μm) and pseudopentaploids (42.742 ± 2.699 μm) between the diploids and pseudohexaploids. The results of our comparison analysis suggested that the width of stomatal GCs differed markedly among different ploidy levels of Chinese chive.

### Comparison of GC chloroplast numbers among different ploidy levels of Chinese chive

**Fig. 6:**
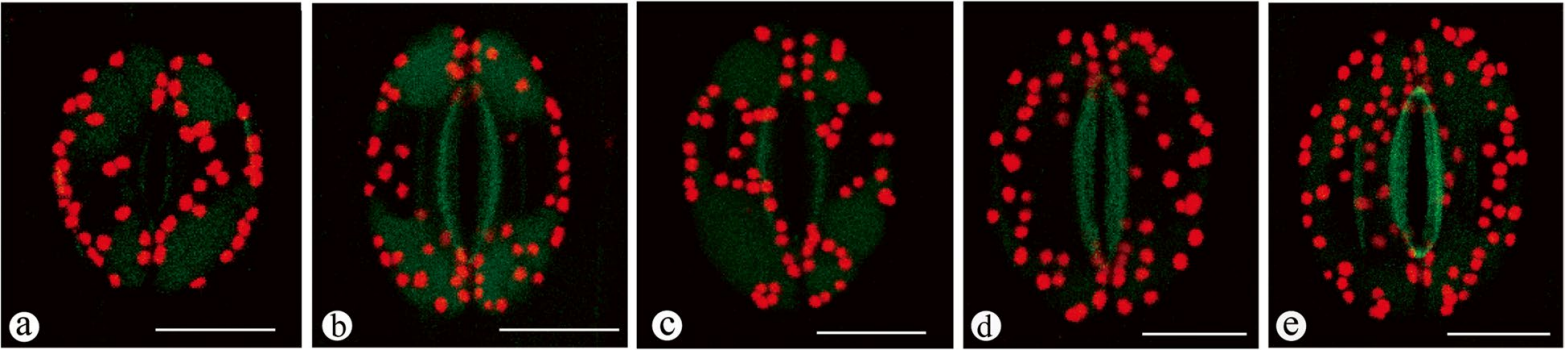
Comparison of chloroplast numbers in the guard cells of Chinese chives with different ploidy levels (scale bar = 20 μm). **a** Chloroplasts in diploids. **b** Chloroplasts in triploids. **c** Chloroplasts in tetraploids. **d** Chloroplasts in pseudopentaploids. **e** Chloroplasts in pseudohexaploids

The GC chloroplast number in Chinese chives with different ploidy levels is shown in Fig. 6. The results showed that the average chloroplast number increased as the ploidy level increased, in which diploids had the fewest chloroplasts (44.64 ± 6.42). The pseudohaxaploids had the most chloroplasts in their GCs (90.23 ± 14.04).

Comparison analysis suggested that the chloroplast number of GCs differed markedly among different ploidy levels of Chinese chives (*p* = *0.000*). Multiple comparison analysis suggested that there was no significant difference between diploids and triploids in terms of chloroplast number, which differed markedly from the results of the other cultivars with different ploidy levels (Table 1).

**Table 1:** Comparison of the chloroplast number in GCs in Chinese chives with different ploidy levels

Different ploidy cultivars	Number of examined	Std. Deviation	Min	Max	Subset for alpha = 0.05			
					1	2	3	4
Diploid	160	6.42	32	60	44.64			
Triploid	293	4.80	24	59	45.84			
Tetraploid	166	5.64	32	66		51.57		
Pseudo-pentaploid	178	7.82	54	94			72.57	
Pseudo-hexaploid	221	14.04	53	128				90.23
Total	1018	20.08	24	128	Sig. = 0.164	Sig. = 1.000	Sig. = 1.000	Sig. = 1.000

However, the average number of chloroplasts per unit area of GCs did not increase as the ploidy level increased, in which pseudohaxaploids had the maximum number of chloroplasts in their GCs (0.041 ± 0.006/μm^2^). Multiple comparison analysis suggested that there was no significant difference between triploids and tetraploids in chloroplast number in the unit area of GCs, which differed markedly from those of the other cultivars (Table 2).

**Table 2:** Comparison of chloroplast numbers per unit area in GCs in different ploidy levels of Chinese chive (150 GCs were chosen randomly for every cultivar)

Different ploidy cultivars	Total area (μm^2^)	Total chloroplast number	Std. Deviation	Subset for alpha = 0.05			
				1	2	3	4
Diploid	199,862.584	6745	0.005	0.034			
Triploid	231,608.204	6827	0.004		0.029		
Tetraploid	266,729.623	7693	0.004		0.029		
Pseudo-pentaploid	296,214.713	10,827	0.005			0.036	
Pseudo-hexaploid	302,828.003	12,578	0.006				0.041
Total	1,297,243.128	44,670	0.007	Sig. = 1.000	Sig. = 0.294	Sig. = 1.000	Sig. = 1.000

## Discussion

In many plant species, stomatal density has often been used as a morphological marker for identifying ploidy level. A negative relation has been observed between stomatal density and ploidy level in some plant species [20, 21, 29]. Lattier et al. [22] reported that measurements of stomatal density revealed a sharp decline in average density from the 4 × cytotype to the 5 × cytotype but no significant difference among the 5x, 6x, and 8 × cytotypes. In our study, anisopolyploids (3 × and 5x) had a higher stomatal density than isopolyploids (2x, 4 × and 6x). In addition, diploids had the lowest rather than the greatest stomatal density, suggesting that stomatal density has no negative relation with ploidy level in Chinese chives.

Some research results have shown that reduced stomatal density results in increased water-use efficiency and drought tolerance [33, 34]. Some researchers have even suggested using genetic manipulation of stomatal density to protect future crop yields [35]. Using the stomatal density to estimate the drought tolerance of plants may be a reliable method. Plants with higher ploidy levels should have a higher drought tolerance if there is a negative relation between stomatal density and ploidy level. This has been confirmed by many plant polyploids having numerous advantages over their diploid relatives, including a greater tolerance for poor soils and drought conditions [36, 37]. For Chinese chives, more work should be done to estimate the environmental tolerance for different ploidy levels. In our study, diploid plants had the lowest density of GCs, which may have resulted in stronger drought resistance. However, this trait is less widely distributed in nature. There may be many reasons for this result, as we have seen the diploid external of Chinese chive (Fig. 3a). These plants may contain more fiber due to the hard leaf blade, which influences the taste, resulting in few people growing it. With respect to Chinese chive, tetraploids are the most widespread in nature. The relatively low density of GCs may have played a part in the process of polyploidization. Chinese chives are cultivated vegetables that are mainly influenced by human activity. Therefore, taste and yield are the main influencing factors considered.

Unlike those of many monocots, the stomata of Chinese chives are formed in defined rows or files along the adaxial and abaxial epidermis, with each stoma consisting of two kidney-shaped GCs. For one species, a higher ploidy level is expected to result in a larger cell size, since it has a higher DNA content. Many studies have shown positive associations between ploidy level and stomatal length [22, 26, 27]. This relationship was also verified in our study. There is some overlap among the different ploidy levels of Chinese chives. There were no significant differences among diploids (21.529 μm), triploids (21.050 μm) and tetraploids (21.589 μm) in the range of variation in the length of GCs (Table 3). However, compared with the other types, pseudopentaploids (25.371 μm) and pseudohexaploids (30.079 μm) showed a greater range of variation in the length of GCs (Table 3). This phenomenon may be associated with the unstable DNA content of pseudopentaploids and pseudohexaploids. For GC width, pseudopentaploids (17.180 μm) and pseudohexaploids (18.970 μm) also had a larger range of variation, while tetraploids had the smallest range (Table 4).

**Table 3:** Comparison of the stomatal length of Chinese chives at different ploidy levels

Different ploidy cultivars	Number of examined	Std. Deviation (μm)	Min (μm)	Max (μm)	Subset for alpha = 0.05				
					1	2	3	4	5
Diploid	1105	3.293	36.223	57.752	44.799				
Triploid	1585	3.000	39.980	61.030		49.319			
Tetraploid	1103	3.476	42.656	64.245			52.151		
Pseudo-pentaploid	1329	3.947	44.363	69.734				56.193	
Pseudo-hexaploid	1095	4.706	44.648	74.727					60.325
Total	6217	6.358	36.223	74.727

**Table 4:** Comparison of the stomatal width of Chinese chives at different ploidy levels

Different ploidy cultivars	Number of examined	Std. Deviation (μm)	Min (μm)	Max (μm)	Subset for alpha = 0.05				
					1	2	3	4	5
Diploid	1105	2.369	27.870	44.616	36.935				
Triploid	1585	2.090	31.570	45.860		37.929			
Tetraploid	1103	1.813	33.507	44.269			39.213		
Pseudo-pentaploid	1329	2.699	34.546	51.726				42.742	
Pseudo-hexaploid	1095	2.756	33.250	52.220					43.281
Total	6217	3.459	27.870	52.220					

**Fig. 7:**
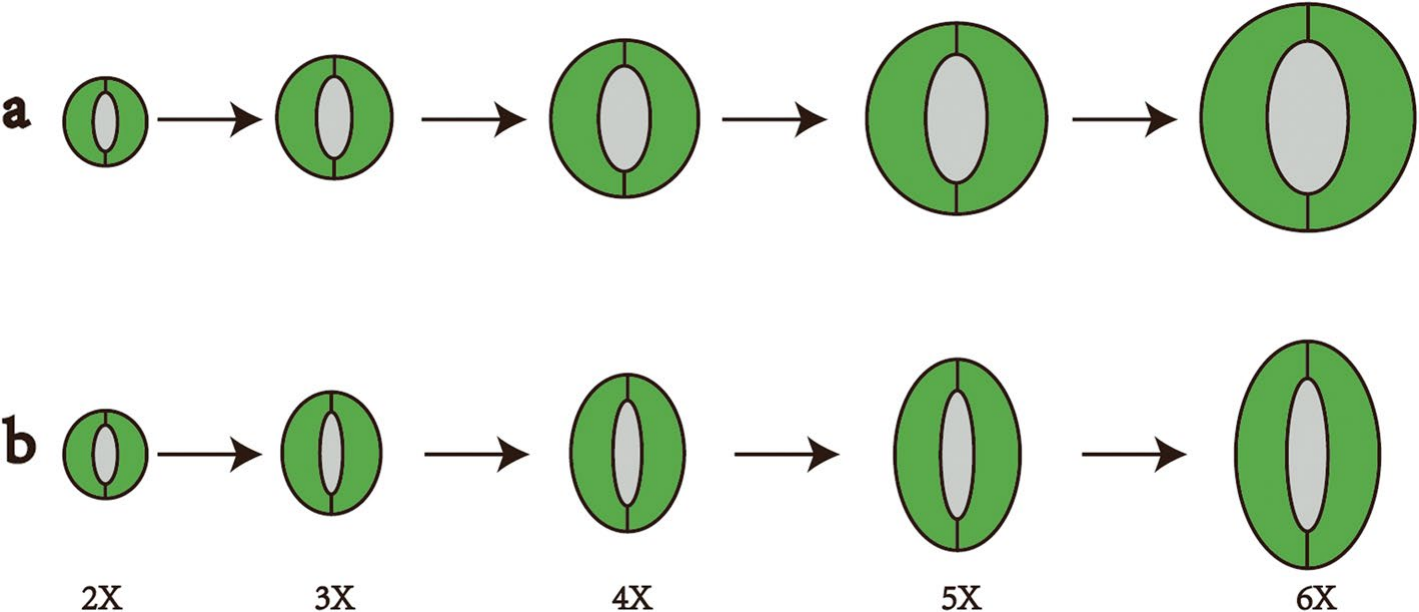
Correlation analyses between the length and width of GCs of Chinese chive cultivars with different ploidy levels. **a** Length and width increased with equal proportion. **b** Length increased more than width

One study showed that not only the length but also the width increased as the ploidy level increased. However, the relationship between length and width is still unclear. In this research, we found that the stomatal length displayed a larger increase than the width as the ploidy level increased, suggesting that the GCs did not increase in equal proportion as the ploidy level increased. Therefore, throughout evolution, stomata do not increase equally in terms of length and width in the process of polyploidization (Fig. 7a). During this process, the length increases more than the width, which results in long and narrow stomata at higher ploidy levels (Fig. 7b). Polyploidy is a major driver of evolution in plants. In their evolutionary history, angiosperms have undergone several polyploidization events [11, 38]. In the process of polyploidization events, GCs become larger but have an elongated structural appearance; this could make stomatal closure and opening efficient, resulting in a higher rate of gas exchange in polyploids. At the same time, this longer and narrow pore structure could effectively prevent the entrance of many foreign materials, such as dust and microorganisms.

Epidermal cells cannot photosynthesize because chloroplasts are not present, but in most plant species, GCs contain photosynthetically active chloroplasts [39–42]. Further studies suggested that the main function of GC chloroplasts is the storage of starch instead of photosynthesis, which influences the balance of the sugar solution for opening GCs [3]. Therefore, the number of GC chloroplasts directly affects the efficiency of the opening and closing of stomata. In our study, the average number of chloroplasts per unit area of GCs did not increase as the ploidy level increased, although the size of GCs increased in this process. It is necessary to pay attention to the efficiency of stomata among these cultivars in a future study. The GC chloroplast number could be used for the detection of hybrids and estimation of ploidy level [43]. Many studies of approximately 80 species, variants and hybrids have revealed that the average GC chloroplast number in leaves or cotyledons ranges from 2.8 to 40.0 for diploids and 5.0 to 73.5 for tetraploids [23, 43]. Furthermore, whole-genome duplication in plants has caused an approximately 1.7-fold increase in GC chloroplast number [24]. In our study, approximately 44.6 and 51.6 chloroplasts were present in the GCs of diploid and tetraploid Chinese chives, respectively (Table 2), suggesting that in Chinese chives, tetraploids could not have originated from the direct duplication of diploids.

## Conclusions

The results of the present study highlight that plants have evolved longer and narrower stomata with more chloroplasts in GCs in the process of polyploidization. This study will provide a reference for the study of stomatal morphological changes during plant evolution. It can also be helpful for the study of the adaptability mechanism of plant polyploids to the environment and the utilization of polyploids.

## Materials and methods

### Materials

A total of 420 cultivars were used in this study, and 10 individuals were chosen randomly for every cultivar. Most cultivars were collected from different regions of China by breeders. All sample collection was permitted by the related department. After years of planting and selecting many collected germplasms, cultivars were formed by division and vegetative propagation. All the materials were located in the resource nursery of the Institute of Agricultural Sciences of Pingdingshan city, Henan Province, China.

### Ploidy analysis by flow cytometry

Every cultivar was derived from the same female parent, and we were confident that the present flow cytometry measurements are reliable and that parthenogenesis was the main method of reproduction.

The ploidy level measurements were conducted using a CyFlow Ploidy Analyzer (Partec, Germany) flow cytometer. 4ˊ,6-Diamidino-2-phenylindole (DAPI) was used as the nuclear dye. A fragment of a young healthy leaf (approximately 20 mg) was chopped using a sharp razor blade. Internal standardization (diploid Chinese chive, 2n = 2x = 16) was chopped together with the detection leaf blades in 800 μL of nuclear extraction solution (45 mM MgCl_2_, 30 mM sodium citrate, 20 mM 4-morpholinepropane sulfonate, 0.1% (v/v) Triton X-100 (pH 7.0) and then filtered through 40 μm nylon mesh after being incubated at room temperature for 3 min. The suspension of released nuclei was stained with 200 μL of DAPI.

### Chromosome counts

The ploidy level of the plantlets was further confirmed by root tip chromosome counting. Excised root tips were incubated in a saturated solution of para-dichlorobenzene for 4 h at 17 °C and then fixed for 24 h in Carnoy’s solution (V_100% ethanol_:V_glacial acetic acid_ = 3:1) at 4 °C. Fixed root tips were hydrolyzed in 1 N HCl for 15 min at 60 °C, rinsed three times with distilled water, stained in carbol fuchsin for 3 min and ultimately crushed under a micro cover glass. At least 20 well-spread metaphase plates per root tip were sampled for chromosome counting.

### Stomatal parameter measurement

Based on the flow cytometry measurements and chromosome counting of different ploidy levels, we further attempted to find differences in stomata. The width and length of the stomata were measured according to the methods of Šmarda et al. [29]. Length of the GCs measured in longitudinal stoma axis, and the width measured as the largest distance between outer GC walls perpendicularly to the longitudinal stoma axis.

In summer 2020 and 2021, different ploidy level cultivars were grown in the same environment in flower pots. Robust mature leaves for each plant were selected to acquire the lower epidermis peel, which could be removed directly from fresh leaves using blade and tweezers. Abaxial and adaxial epidermal tissues were removed simultaneously due to the narrow leaf bleed of the Chinese chive. The removed epidermis peel was unfolded completely using distilled water on microscope slides. Each slide represented a random leaf sample and was treated as a replicate for further analysis.

### Statistics and data analysis

All preparations were viewed using a confocal laser scanning microscope (LSM; Carl Zeiss 880, Germany). Stomatal density was observed by the z-stack scan technique using 405 nm excitation light. Images were captured randomly and processed using image analysis software (ZEN 3.1 Blue Edition, Zeiss, Germany). More than 20 epidermal peels were obtained from every selected material. A minimum of 1000 stomata for every material were measured for GC length and width. A total of 150 GCs were chosen randomly for area measurement for every cultivar. All data were measured via digital image control (DIC) with an LSM. The size (length and width) and area of GCs were measured using distance and contour measurement tools respectively (ZEN 3.1 Blue Edition, Zeiss). Chloroplast GC pairs were also observed by the z-stack scan technique using excitation at 514 nm.

A comparison of the data, including stomatal density and GC length and width, was compared by the Student Newman Keuls Test (S–N-K) method among different ploidy levels. The statistical data were analyzed using IBM SPSS Statistics 19 (IBM, Armonk, NY, USA). GC length and width were also compared, as shown in boxplots using Origin 2019 (OriginLab, Northampton, Massachusetts, USA).

### Supplementary Information

**Additional file 1.** Chloroplast number of guard cells in different ploidy levels of Chinese chives.

**Additional file 2. **Stomatal number in the area of 850.2μm × 850.2μm of epidermal layer in different ploidy levels of Chinese chives.

**Additional file 3.** Stomatal length of different ploidy levels of Chinese chives.

**Additional file 4.** Stomatal width of different ploidy levels of Chinese chives.

## Data Availability

All the data are available in the submitted manuscript.

## Abbreviation

GC: Guard cell
